# Enhancing Knowledge Retention by Simulation-Based Learning Among First-Year Medical Students

**DOI:** 10.7759/cureus.89657

**Published:** 2025-08-08

**Authors:** Nimarpreet Kaur, Bhupendra Yadav, Deepti Dwivedi, Harminder Kaur, Pragyashaa Chaudhary

**Affiliations:** 1 Physiology, Faculty of Medicine and Health Sciences, SGT University, Gurugram, IND; 2 Simulation, The National Reference Simulation Centre, Faculty of Medicine and Health Sciences, SGT University, Gurugram, IND; 3 Physiology, ESIC Hospital and PGIMSR, Basaidarapur, New Delhi, IND; 4 Physiology, SGT University, Gurugram, IND

**Keywords:** cardiovascular physiology modules, competency based medical education (cbme), objective structured practical exam (ospe), satisfaction index, simulation-based learning (sbl)

## Abstract

Introduction

Simulation-based training has been a vital part of medical education since Competency-Based Medical Education (CBME) was introduced, and new guidelines since 2023 have expanded to include simulation as a mandatory methodology of teaching. This method enables learners to build and develop both technical and non-technical abilities in a safe and controlled setting, enhancing their preparedness for real-life medical scenarios. Simulation-based training improves skill acquisition and retention and enhances learners' confidence, reduces anxiety, reinforces learning, corrects errors, and promotes reflective practice, in contrast with the traditional method of teaching. This, in turn, creates an immersive and interactive environment.

Methods

The research project involved 150 first-year MBBS students at NRSC and the Department of Physiology. The CVS modules were prepared, and the sensitization of students as well as the faculty was done at the simulation lab for orientation before the implementation of the module. After the module creation and content validation, all 150 students were taught in a traditional way in five classes covering the modules, and a multiple-choice question (MCQ) test was conducted. Then the students were divided into small groups of 20 each and sent to the simulation lab, and the modules were taught in five days. Pre- and post-tests were conducted, and feedback regarding the SBT was collected on the last day of the SBT. A satisfaction survey was also carried out at the end of the training, and the satisfaction index (SI) was calculated and presented in graphs. Transcripts were prepared for open-ended questions in feedback and subjected to thematic analysis to derive themes and codes.

Results

The mean pre-test objective structured practical examination (OSPE) scores were 7.27 ± 1.29, and the post-test OSPE scores were 12 ± 1.64, and the difference was statistically significant. The MCQs were based on factual knowledge, concepts, and communication. A significant improvement in the scores was observed, as the lowest scores increased from 4 to 11 and the highest scores increased from 9 to 20. Additionally, the feedback was obtained on a Likert scale from both the students as well as the faculty members. Over a range of 1-100, the maximum SI of 92.8 for the statement "simulation-based learning is a useful learning strategy" indicates that this type of learning should be part of the curriculum; also, the minimum SI of 81.6 for the statement "the training session resembles a real life situation" is due to difficulty in making a connection with the simulators. Regarding faculty perception, on assessing the SI among the faculty, it was found that the highest SI was associated with the statement that "the simulation lab is a good source of information", and the only difficulty observed among the faculty was in the preparation of modules and teaching material, as reflected by SI 61.7. Overall, simulation-based learning (SBL) offers an avenue for hands-on, experiential learning in a safe and regulated clinic setting.

Conclusions

SBL can provide a wide range of practice opportunities and offer one of the most effective learning methods in higher education. This works well for beginners and helps in the development of critical thinking and concept clarity. SBL is a useful tool that should be incorporated from the first year and enhances the clinical orientation of the students in studying basic sciences.

## Introduction

Medical education is undergoing a paradigm shift to address the long-standing disconnect between basic science instruction and clinical practice. Traditionally, first-year medical students are introduced primarily to foundational subjects with minimal or no exposure to clinical environments, resulting in a theoretical understanding that lacks real-world application. Simulation-based learning (SBL) has emerged as a powerful pedagogical tool to bridge this gap by providing experiential learning opportunities that integrate cognitive, psychomotor, and affective domains. Studies have shown that SBL not only enhances students’ medical knowledge and technical proficiency but also improves communication, teamwork, and decision-making skills within realistic clinical scenarios [1,2].

The pedagogical structure of SBL aligns closely with Peyton’s four-step approach to clinical skills training - comprising demonstration, deconstruction, comprehension, and performance - thereby offering a systematic framework for skill acquisition [3,4]. For example, simulation using high-fidelity patient mannequins in cardiac physiology is more effective than traditional didactic methods [5]. Similarly, Daniel et al. have demonstrated that simulation significantly enhances both cognitive understanding and hands-on performance [6]. Building on this foundation, the current study was designed to assess the effectiveness of an integrated simulation scenario aimed at first-year medical students. The simulation incorporated essential components such as intellectual engagement, technical skills, communication, and team-based tasks. This study specifically aims to evaluate whether SBL can serve as a beneficial adjunct to conventional teaching methods and to explore students’ perceptions of its impact on their early medical education [7].

Ultimately, this study aims to develop a module on simulation along with the blueprint for evaluation in teaching MBBS students. With the incorporation of simulation in the teaching methodology, the study has a primary focus on the measurement of improvement in academic performance. Hence, the focus of our study is to answer the following questions: Does simulation provide a more immersive experience than traditional lectures? Does integrating simulation methodology with traditional lectures improve knowledge acquisition? Lastly, is simulation effective and beneficial for all students in enhancing concept understanding?

## Materials and methods

Data collection

**Figure 1:**
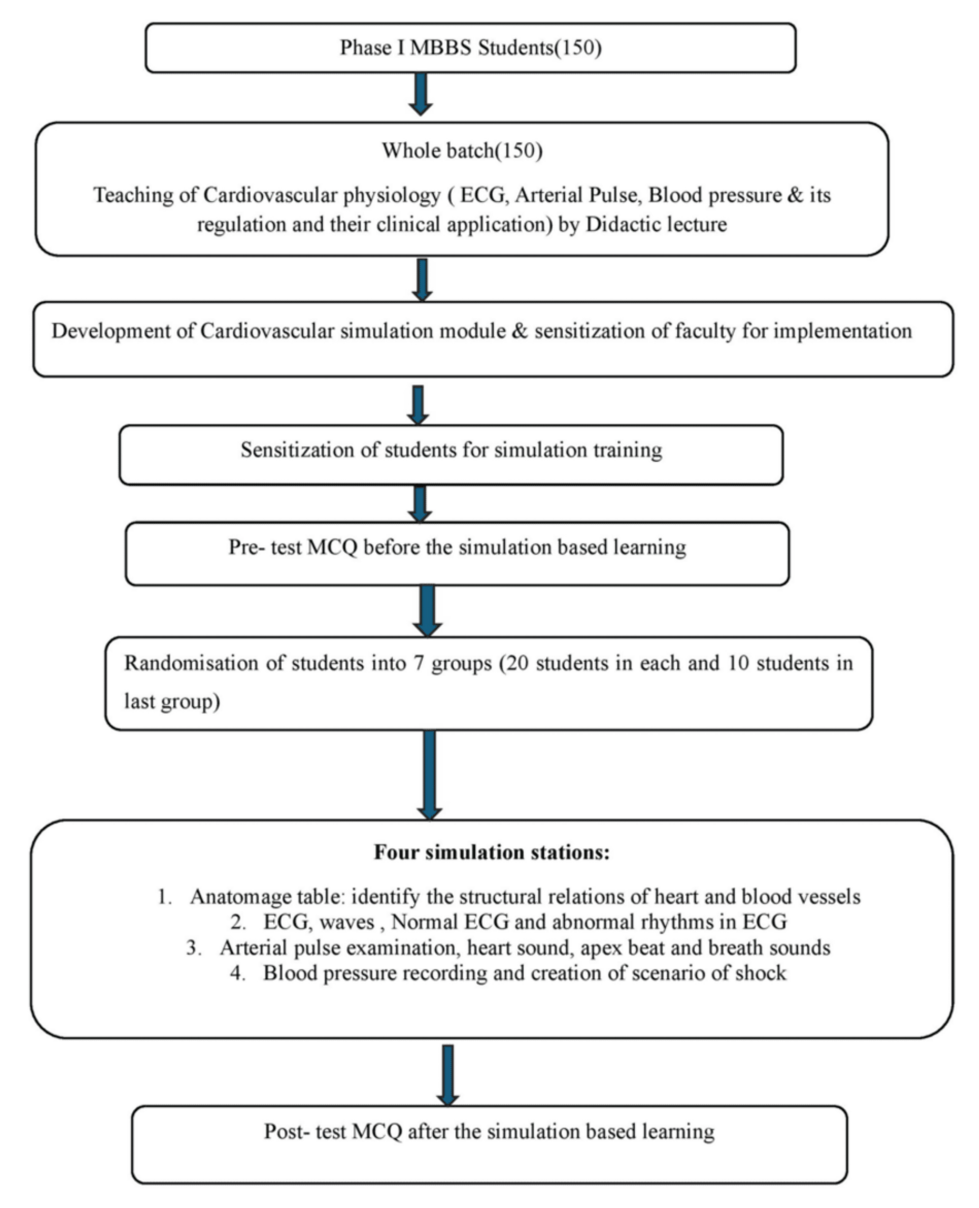
Concept map ECG: electrocardiogram; MCQ: multiple-choice question

This cross-sectional observational study (pre- and post) involved 150 first-year MBBS students at the NRSC and the Department of Physiology at the Faculty of Medicine and Health Sciences, SGT University, Gurgaon, after obtaining the Institutional Ethics Committee approval (No. IEC/F/2025-13). The students were allocated into seven groups (20 students each and 10 students in the last group) and were conducted over five months: from September 2024 to January 2025 (Figure 1). The study tools were as follows: pre- and post-objective structured practical examination (OSPE) scores, feedback from faculty and students, and satisfaction surveys, as illustrated in Table 1.

**Table 1:** Tool development

Traditional method	Simulation training	Post-simulation training
PowerPoint presentation	15 min written pre-test	Satisfaction survey: faculty and students
Video clips	15 min briefing session	MCQ assessment based on factual knowledge, conceptual knowledge, and communication
Reading material	30 min simulation exercise	
	40 min debriefing session	
	15 min post-test feedback	

Evaluation

20 MCQs were prepared for the exam on three parameters: factual knowledge, concept knowledge, and communication, to assess the academic performance. A feedback questionnaire for students’ perception regarding simulation training was designed and validated. It comprised 12 questions, out of which 10 questions were on a Likert scale from 1 to 5, and five questions were open-ended, out of which two questions were thematically analyzed. Feedback was taken immediately after the simulation lab completion for every batch on the fifth day. Feedback from the faculty members was taken post-sensitization on the Likert scale.

Data analysis

Quantitative Data

OSPE scores and exam scores were compared with the paired t-test to assess the improvement in academic performance. Collected data was entered into Microsoft Excel and SPSS Statistics version 26 (IBM Corp., Armonk, NY). Mean, standard deviation (SD), p-value, minimum and maximum values, and range of participants’ scores were computed. Student’s t-test was used to compare the mean scores of the students. A p-value <0.05 was considered statistically significant.

Feedback Responses on Likert Scale: Descriptive Statistics

Satisfaction index (SI) and median values: Students' feedback responses were analyzed in terms of proportions and SI. SI was calculated for the 10 items answered by students on the 5-point Likert scale (1=strongly disagree, 2=Disagree, 3=Neutral, 4= Agree, and 5=Strongly Agree).

SI was calculated for each item of the questionnaire by adopting the following formula [8], which states that

S I = {(a X1)+ (b X2)+ (c X 4)+ (d X 5)} X 20 divided by N

Where n1, n2, n4, and n5 = number of students who marked the response 1, 2, 4, and 5 on the Likert scale, respectively; N = total number of students who participated.

Thematic analysis: Two items of the questionnaire, which were open-ended ended were subjected to thematic analysis, and relevant themes and codes were prepared.

## Results

After the module creation and content validation, the whole batch of 150 students was taught in a traditional way in five classes covering the modules. Then the students were divided into small groups of 20 each and sent to the simulation lab as scheduled in the timetable, and there they were taught the five modules in five days. The mean test score of pre-OSPE was 7.27 ± 1.29, while it was 12 ± 1.64 post-OSPE, and the difference was statistically highly significant as per the paired t-test. 

The academic performance was assessed using 20 MCQs, which included nine factual knowledge-based questions, eight conceptual knowledge-based questions, and three communication-based questions as depicted in Table 2. The mean pre-test score based on factual knowledge was 2.75 ± 0.82, and the post-test score was 6.65 ± 0.98; the difference was statistically significant. The mean pre-test score of questions based on conceptual knowledge was 3.42 ± 1.03, and post post-test score was 4.51 ± 1.20, and the difference was found to be statistically significant. The mean pre-test score of questions based on communication was 1.09 ± 0.36, and post post-test score was 1.38 ± 0.69, and the difference was statistically significant. On statistical analysis using the two-tailed paired t-test, the difference in mean between pre- and post-score was found to be statistically highly significant in all three parameters.

**Table 2:** The comparison of scores per different assessment parameters P-values were calculated using the paired t-test. Results were considered significant at p<0.05 SD: standard deviation

Question	Pre-test score, mean ± SD	Post-test score, mean ± SD	t-value	P-value
Factual knowledge (9)	2.75 ± 0.82	6.65 ± 0.98	37.38	<0.001*
Conceptual knowledge (8)	3.42 ± 1.03	4.51 ± 1.20	8.44	<0.001*
Communication (3)	1.09 ± 0.36	1.38 ± 0.69	4.56	<0.001*

As shown in Table 3, in the pre-test, out of 150 students, four (2.67%) scored less than 20% of the marks, 23 (15.33%) scored between 21% and 30%, 84 (56%) scored between 31% and 40%, and 39 (26%) scored between 41% and 50%. In the post-test, all the students scored above 50% of the marks; of them, 87 (58%) students scored between 51% and 60% of the marks, 44 (29.33%) students scored between 61% and 70%, 13 (8.67%) students scored between 71% and 80%, three (2%) students scored between 81%-90%, and 3 (2%) students scored between 91% and 100% (Table 3).

**Table 3:** Comparison of academic performance pre- and post-simulation training

Percentage of marks (0-20)	Range of marks	Pre-test, n (%)	Post-test, n (%)
Less than 20%	3-4	4 (2.67%)	0
21%-30%	5-6	23 (15.33%)	0
31%-40%	7-8	84 (56%)	0
41%-50%	9-10	39 (26%)	0
51%-60%	11-12	0	87 (58%)
61%-70%	13-14	0	44 (29.33%)
71%-80%	15-16	0	13 (8.67%)
81%-90%	17-18	0	3 (2%)
91%-100%	19-20	0	3 (2%)

**Figure 2:**
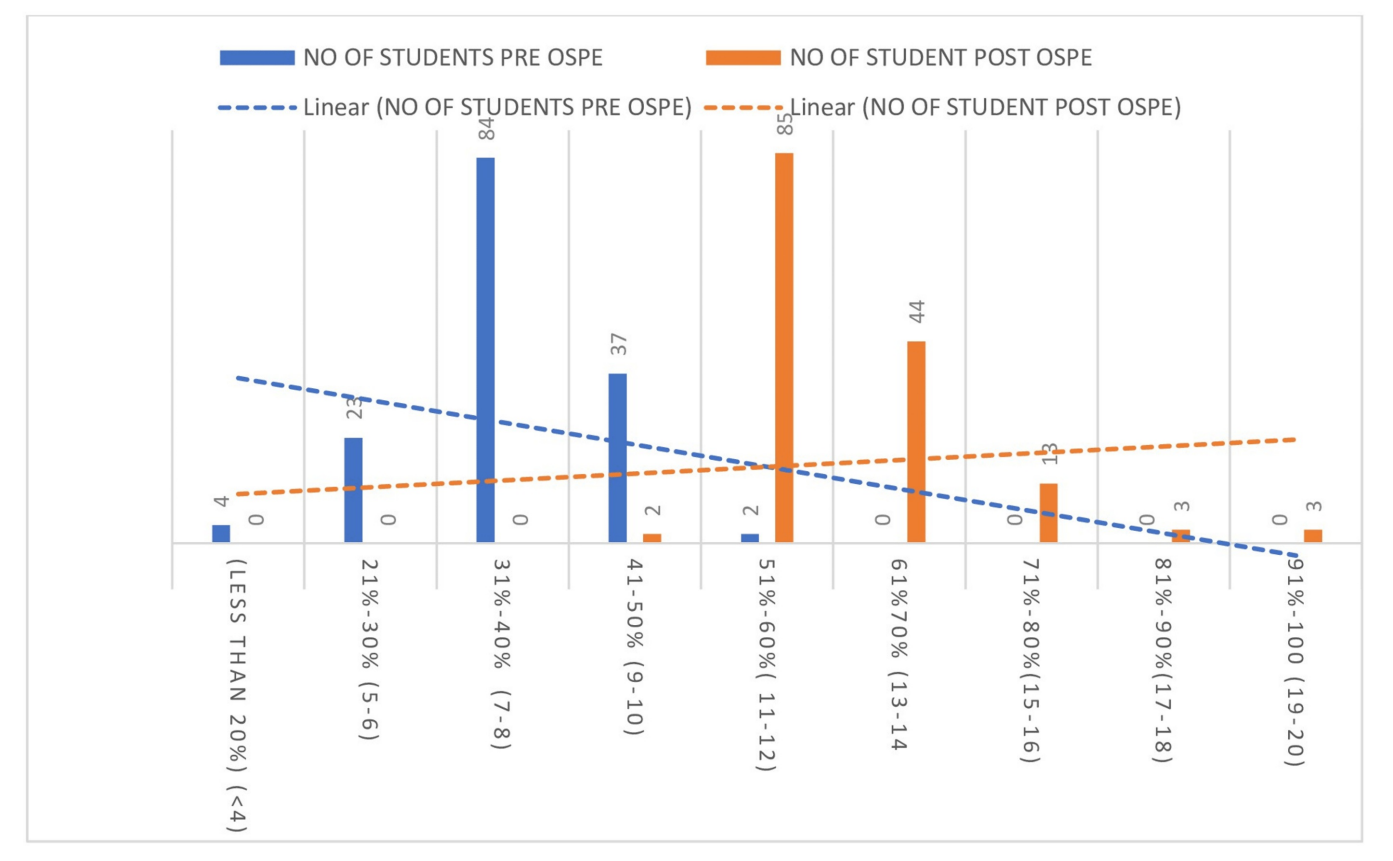
Comparison of academic performance pre- and post-simulation training The bar graph depicts the number of students across various score ranges: pre-OSPE (blue bars); post-OSPE (orange bars) OSPE: objective structured practical examination

As illustrated in Figure 2, there was a marked shift toward higher score categories following simulation training. The linear trend lines (dashed) highlight a downward trend in low scores pre-training and an upward trend in performance post-training, indicating an overall improvement in academic performance after the intervention.

As presented in Table 4, over a range of 1-100, the maximum SI of 92.8 obtained for the statement that "simulation based learning is a useful learning strategy" indicates the fact that this type of learning should be part of the curriculum; the minimum SI of 81.6 for the statement that "the training session resembles a real life situation" is due to difficulty in making a connection with the simulators.

**Table 4:** Students' feedback on Likert scale (SD=1 to SA=5) and satisfaction index SD: strongly disagree; D: disagree; N: neutral; A: agree; SA: strongly agree; SI: satisfaction index

Statement	SD	D	N	A	SA	SI
The training session resembled a real-life situation	0 (0.0%)	1 (0.67%)	12 (8%)	75 (50%)	62 (41.33%)	81.6
Cardiovascular physiology concepts were easily learned by simulation	0 (0.0%)	0 (0.0%)	6 (4%)	92 (61.33%)	52 (34.67%)	83.73
Small group teaching with simulation is a better way of teaching/learning	0 (0.0%)	0 (0.0%)	7 (4.67%)	52 (34.67%)	91 (60.66%)	88.4
Simulation can improve effective participation and communication	0 (0.0%)	0 (0.0%)	1 (0.67%)	58 (38.67%)	91 (60.66%)	91.6
I would prefer to have more simulation-based teaching in addition to traditional teaching methods in learning	0 (0.0%)	0 (0.0%)	2 (1.33%)	47 (31.33%)	101 (67.34%)	92.4
Simulation-based learning is a useful learning strategy	0 (0.0%)	0 (0.0%)	0 (0.0%)	54 (36%)	96 (64%)	92.8
Simulation-based learning made the subject more Interesting	0 (0.0%)	0 (0.0%)	3 (2%)	51 (34%)	96 (64%)	91.2
Simulation-based learning helped me to apply what I learnt	0 (0.0%)	0 (0.0%)	4 (2.67%)	77 (51.33	69 (46%)	87.0
Simulation-based learning helped me retain knowledge	0 (0.0%)	0 (0.0%)	5 (3.33%)	76 (50.67%)	69 (46%)	86.5
Simulation-based learning developed clinical decision-making	0 (0.0%)	0 (0.0%)	7 (4.67%)	63 (42%)	80 (53.33%)	86.9

Various themes and codes that emerged in response to the first open-ended question, “Opinion about the methodology of training,” are elaborated in Table 5.

**Table 5:** Thematic analysis of responses to the first open-ended question

Themes	Codes/categories
Innovative and unique	Good methodology
	Better experience of practical
	Innovative method of training
	Unique method
	Best way to learn
Inculcate clinical reasoning skills	Hands-on experience
	Engaging
	Enhances clinical skills
	Amazing
	Concept clear
Enhancing clinical skills	Application of concepts
	Better clinical correlation
	Safe to practice. No danger
	Repetition
Improving conceptual clarity	Concepts are clear and stronger
	Thorough clarity about concepts
	Emphasis on topics required in clinical practice
	Understands concepts from a new perspective

The themes and codes that emerged in response to the second open-ended question, “How confident are you about using the training methodologies learnt?” are presented in Table 6.

**Table 6:** Thematic analysis of responses to the second open-ended question

Themes	Codes/categories
Innovative and unique	Good methodology
	Better experience of practical
	Innovative method of training
Improves in-depth learning	Unique method
	Best way to learn

**Figure 3:**
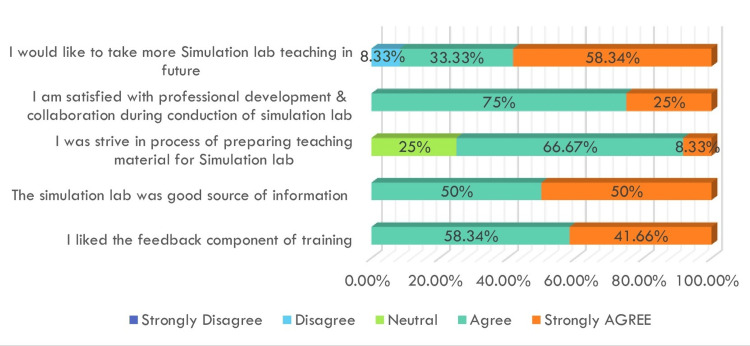
Faculty feedback on simulation-based teaching using a Likert scale

The horizontal bar chart in Figure 3 represents faculty responses to various aspects of simulation training. A majority expressed agreement or strong agreement with the feedback component (58.34% strongly agreed), the simulation lab as a source of information (50% agreed), and the value of professional development (75% agreed). Most faculty found the experience of preparing teaching material valuable and indicated interest in conducting future simulation sessions. These findings reflect a generally positive perception of simulation-based education among faculty.

**Figure 4:**
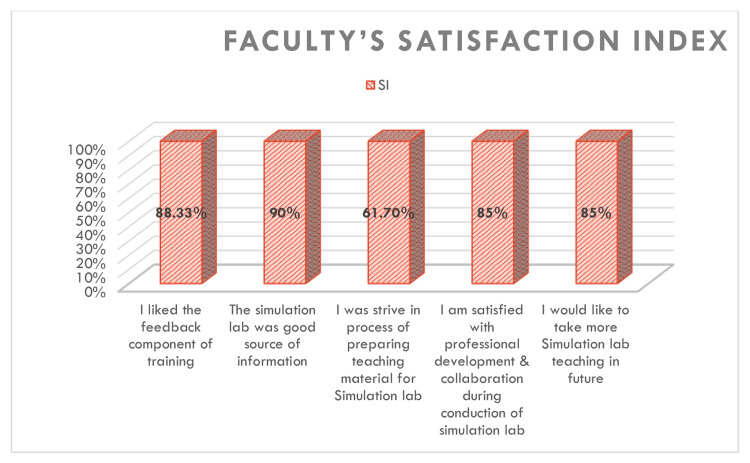
Faculty’s SI regarding simulation-based teaching SI: satisfaction index

The vertical 3D bar chart in Figure 4 shows the SI rates for different aspects of the simulation experience. Faculty reported high satisfaction with the feedback component (88.33%), the simulation lab as an informative resource (90%), and collaboration and professional development (85%). Satisfaction was comparatively lower (61.7%) for the preparation of teaching materials. The overall trend indicates a strong endorsement of simulation-based teaching by faculty.

## Discussion

Simulation-based learning offers a learning experience with the approximation of practice, allows limitations of learning in real-life situations to be overcome, and can be an effective approach to developing complex skills. Beaubien and Baker (2004) defined simulation as a tool that reproduces the real-life characteristics of an event or situation. Cook (2013) further stated that simulation is an “educational tool or device with which the learner physically interacts to mimic real life” and in which they emphasize “the necessity of interacting with authentic objects” [9,10].

SBL resulted in the enhancement of knowledge about clinical examination of the cardiovascular system in our study, as evidenced by the significant difference in mean test scores. The present study findings align with those by Bray et al., who used a pretest and post-test to assess the impact of simulation-based learning on pharmacy students [11]. Our results are also comparable to those of Zarifsanaiey et al., who conducted an OSCE among 40 students to assess the effectiveness of the traditional method over simulation-based learning. They found significantly higher performance in terms of OSCE scores in the simulation-based learning group [12]. The possible reason for the improvement in the post-test scores could be that simulation enhances active learning among students by improving their understanding and knowledge.

An in-depth analysis of the students was further performed based on the type of questions asked in the exams to study the improvement in academic performance; it was found that there was significant improvement in mean post-test scores. When the comparison of academic performance pre- and post-simulation training was plotted percentage-wise, it was found that there was a total reversibility of marks after the simulation learning, indicating that learning with the help of simulation-based activities can help the students to link basic science concepts to the practical applications (Table 2, Figure 2) [13]. Our study results are concordant with the study by Jabaay et al. in which the students’ performance improved by 18% following the simulation experience [14]. The lowest score increased from 4 to 11, and the highest score increased from 9 to 20 (Table 3), indicating that simulation training improves retention of knowledge and enhances learners' confidence and reduces anxiety, as supported by other studies by various authors [15]. Our findings demonstrated students’ perception of patient simulators and their usefulness; they reported that SBL, as an addition to learning with real patients, improves retention of learning material, enhances decision-making skills, and provides a conducive learning environment. The results are supported by other authors, including Traynor et al [16] and Ennen and Satin [17], who also reported that students acquire knowledge and confidence in the clinical setting through simulation workshops, which also increase communication and teamwork in emergencies.

Themes emerging from open-ended feedback questions were documented (Tables 5, 6), which included the exposure to a semirealistic environment and opinion about the methodology of training, such as knowledge retention, concept building, working in collaboration, and in a nonthreatening learning environment. The responses given by the students highlighted the key features of SBL for teaching clinical skills: good methodology, better experience, unique method, innovative method, enhances skills, clarifies the concepts, confidence to tackle real-life situations, and increases the limits of thinking. Regarding faculty perception and SI, it was found that the maximum satisfaction index was associated with the statement "simulation lab is a good source of information," and the only difficulty observed among the faculty was in the preparation of modules and teaching material, as reflected by an SI of 61.7 (Figure 4).

Based on our findings, we conclude that the medical graduates require sufficient exposure to situations involving real patients for learning the mandatory skills. For this, there should be accountability for the patient's security, convenience, and well-being. These are two distinct requirements that present a conundrum in medical education [18]. Thus, simulation-based learning can help students expand their expertise, skills, and mindsets while also sparing patients from undue suffering and hazards. Simulation-based learning can be used to navigate ethical issues and solve practical dilemmas [19].

Limitations

This study has a few limitations. One major limitation lies in the fact that not all students went through the pre- and post-test at the same point in time, as the study was conducted over several days and different slots. Secondly, sensitization of the faculty and students was a big challenge while conducting the study. Thirdly, the module preparation by the faculty proved to be a tedious task due to time constraints and led to dissatisfaction among a few faculty members in terms of its implementation.

## Conclusions

In higher education, simulation-based learning is one of the best approaches and can offer an extensive array of practice opportunities. It is a useful tool that should be implemented from the first year itself, and it provides students with clinical orientation while studying basic sciences. It works well for novices and helps in the development of critical thinking and concept clarity. Strategic planning, faculty development, and integrating SBL with curriculum objectives will have a significant impact on the skill development among medical students from the inception of medical training itself.
